# Farewell to Bright-Line: A Guide to Reporting Quantitative Results Without the S-Word

**DOI:** 10.3389/fpsyg.2020.00815

**Published:** 2020-05-13

**Authors:** Kevin M. Cummins, Charles Marks

**Affiliations:** ^1^Division of Infectious Disease and Global Public Health, SDSU-UCSD Joint Doctoral Program in Interdisciplinary Research on Substance Use, San Diego, CA, United States; ^2^Department of Psychology, University of California, San Diego, San Diego, CA, United States; ^3^Department of Psychiatry, University of California, San Diego, San Diego, CA, United States

**Keywords:** scientific communication, statistical significance, null hypothesis significance testing, confidence intervals, bright-line testing

## Abstract

Recent calls to end the practice of categorizing findings based on statistical significance have focused on what not to do. Practitioners who subscribe to the conceptual basis behind these calls may be unaccustomed to presenting results in the nuanced and integrative manner that has been recommended as an alternative. This alternative is often presented as a vague proposal. Here, we provide practical guidance and examples for adopting a research evaluation posture and communication style that operates without bright-line significance testing. Characteristics of the structure of results communications that are based on conventional significance testing are presented. Guidelines for writing results without the use of bright-line significance testing are then provided. Examples of conventional styles for communicating results are presented. These examples are then modified to conform to recent recommendations. These examples demonstrate that basic modifications to written scientific communications can increase the information content of scientific reports without a loss of rigor. The adoption of alternative approaches to results presentations can help researchers comply with multiple recommendations and standards for the communication and reporting of statistics in the psychological sciences.

## Introduction

The abandonment of significance testing has been proposed by some researchers for several decades (Hunter, 1997; Krantz, 1999; Kline, 2004; Armstrong, 2007). In place of heavy reliance on significance testing, a thorough interrogation of data and replication of findings can be relied upon to build scientific knowledge (Carver, 1978) and has been recommended in particular for exploratory research (Gigerenzer, 2018). The replication crisis has demonstrated that significance testing alone does not ensure that reported findings are adequately reliable (Nosek et al., 2015). Indeed, the practice of focusing on significance testing during analysis is a motivator for “P-hacking” and expeditions into the “garden of forking paths” (Gelman and Loken, 2014; Szucs and Ioannidis, 2017). These problems are compounded by the outright misunderstanding and misuse of *p*-values (Goodman, 2008; Wasserstein, 2016). The practice of bright-line significance testing has been considered a generator of scientific confusion (Gelman, 2013), though appropriately interpreted *p-value*s can provide guidance in results interpretation.

A recent issue of *The American Statistician* and a commentary in *Nature* suggest a seemingly simple conciliatory solution to the problems associated with current statistical practices (Amrhein et al., 2019; Wasserstein et al., 2019). The authors of these articles advocate ending the use of bright-line statistical testing in favor of a thoughtful, open, and modest approach to results reporting and evaluation (Wasserstein et al., 2019). This call to action opens the door to the widespread adoption of various alternative practices. A transition will require authors to adopt new customs for analysis and communication. Reviewers and editors will also need to recognize and accept communication styles that are congruent with these recommendations.

Many researchers may subscribe to the ideas behind the criticisms of traditional significance testing based on bright-line decision rules but are unaccustomed to communicating findings without them. This is a surmountable barrier. While recent calls to action espouse principles that researchers should follow, tangible examples, both of traditional approaches to statistical reporting, as well as the newly recommended ones, may serve as a needed resource for many researchers. The aim of this paper is to provide researchers guidance in ensuring their repertoire of approaches and communication styles include approaches consistent with these newly reinforced recommendations. Guidance on crafting results may facilitate some researchers’ transition toward the execution of the recent recommendations.

### What Is the Dominant Approach?

Null hypothesis significance testing (NHST) dominates the contemporary application of statistics in psychological sciences. A common approach to structuring a research report based on NHST (and we note that there are many variations) follows these steps: *first*, a substantive question is articulated; *then*, an appropriately matched statistical null hypothesis is constructed and evaluated. The statistical question is often distinct from the substantive question of interest. Next, upon execution of the given approach, the appropriate metrics, including the *p*-value, are extracted, and, *finally*, if this *p*-value (or equivalently the test statistic) is more extreme than a pre-determined bright-line α, typically 0.05, a declaration that the result is significant is issued.

The dominant style of research communication that has arisen from this approach has emphasized the dissemination of findings that meet the significance threshold, often disregarding the potential for non-significant findings to provide some utility in addressing substantive scientific questions at hand. The concern with dichotomizing findings was distilled by Altman (1990) when he wrote, “It is ridiculous to interpret the results of a study differently according to whether or not the *P*-value obtained was, say, 0.055 or 0.045. These should lead to very similar conclusions, not diametrically opposed ones.” Further, this orientation has facilitated the de-emphasis of the functional associations between variables under investigation. In the simplest case, researchers have failed to focus on the association magnitude (Kirk, 1996). Whereas Kelley and Preacher (2012) have described the differences between effect size and effect magnitude, we propose a more general focus on the functional associations between our variables of interest, which are often complex, contingent, and curvilinear, and so often cannot be adequately distilled into a single number. Although we will refer to effect sizes using the conventional definition, we want the reader to recognize that this usage is not consistently tied to causal inference, in practice. Adapting Kelley and Preacher’s (2012) definition, we treat effect size as the quantitative reflection(s) of some feature(s) of a phenomenon that is under investigation. In other words, it is the quantitative features of the functional association between variables in a system under study; this tells us how much our outcome variable is expected to change based on differences in the predictors. If the outcome variable displays such small changes as a result of changes in a predictor that the variance is of little practical value, a finding of statistical significance may be irrelevant to the field. The shape, features, and magnitude of functional associations in studied phenomena should be the focus of researchers’ description of findings. To this end, the reader is encouraged to consult several treatments of effect size indices to assist in the identification of appropriate statistics (Ellis, 2010; Grissom and Kim, 2014; Cumming, 2017).

Herein, we present a generalized version of this significance orientation communication style (SOCS), steps that can be taken to transition to a post-significance communication style (POCS) that will facilitate researchers’ focus on the structure of the associations they are studying rather than just evidence of an association. Examples of how SOCS results write-ups may be updated to meet the standards of this new style follow.

### Significance Orientation Communications Structure

The structure for a passage in a results section written in the SOCS frequently includes:

1. A reference to a table or figure,
2. A declaration of significance, and,
3. A declaration of the direction of the association (positive or negative).

The ordering is not consistent but often begins with the reference. The authors write a statement such as, “Table 1 contains the results of the regression models,” where Table 1 holds the statistics from a series of models. There may be no further verbal description of the pattern of findings. The second sentence is commonly a declaration of the result of a significance test, such as, “In adjusted models, depression scores were significantly associated with the frequency of binge drinking episodes (*p* < 0.05).” If the direction of association was not incorporated into the second sentence, a third sentence might follow; for instance, “After adjustment for covariates, depression scores were positively associated with binge episodes.” Variation in this structure occurs, and in many instances, some information regarding the magnitude of associations (i.e., effect sizes) is presented. However, because this approach focuses on the results of a significance test, the description of the effect is often treated as supplemental or perfunctory. This disposition explains many misunderstandings and misuses of standardized effect sizes (Baguley, 2009). Findings that do not meet the significance threshold are often only available to the reader in the tables and frequently not considered when answering the substantive question at hand. However, interval estimates can and should be leveraged even when the null hypothesis is not rejected (Kelley and Preacher, 2012).

### Post-significance Communications Structure

Here, we present an overarching structure for what text in the results could look like when using post-significance communications structure (POCS). The emphasis shifts from identifying significant results to applying all findings toward the purpose of answering the substantive question under study. The **first sentence** can be considered a direct answer to this question, which the authors proposed in the introduction – the findings of the statistical tests should be placed in the context of the scientific hypotheses they are addressing. **Next,** the quantitative results of the statistical analyses should be described, and, as a part of this description, a directional reference to supporting tables and figures can be noted. Emphasis should be placed on making sure the results are presented in a form that allows the reader to confirm if the author’s assessment in the first sentence is appropriate. This will often include a parenthetical notation of the *p*-value associated with the presented parameter estimates. The significance is not an isolated focus and its presentation is not contingent on the *p*-value reaching a threshold. Instead, *p*-values are part of the support and context for the answer statement (Schneider, 2015). This is reflected in their position within the paper. They can be placed in tables, presented parenthetically, or set off from the rest of the text through the use of commas when parentheses would add an additional level of enclosure. *P*-values should always be presented as continuous statistics and recognized as providing graded levels of evidence (Murtaugh, 2014; Wasserstein et al., 2019).

Even where *p*-values are large, the authors should focus on describing patterns relevant to the question at hand. Assuming a good study design, the best estimate, based on the data being presented, are the point estimates, regardless of the *p*-value. Considering the context of the interval estimates is also critical in all circumstances because we do not want to conflate random noise with effects. The **remaining sentences** should be descriptions of the auxiliary patterns in the data that are pertinent to the scientific questions at hand. In many cases, these descriptions function as annotations of the key patterns found in the tables and figures.

To help clarify how we can transition from the SOCS style to the POCS style, we provide two examples from our own research.

#### Example: Factors Related to Injection Drug Use Initiation Assistance

Using data from a multi-site prospective cohort study, we investigated factors that were associated with providing injection assistance to previously injection-naïve individuals the first time they injected (Marks et al., 2019). Most initiations (i.e., the first time an injection-naïve person injects drugs) are facilitated by other people who inject drugs (PWID). There is evidence that PWID receiving opioid agonist treatment have a reduced likelihood of providing assistance to someone initiating injection drug use (Mittal et al., 2017). We are interested in understanding the extent to which opioid agonist treatment enrollment and other factors are associated with assisting injection drug use initiation. The following describes part of what we recently found, using a conventional SOCS approach (Marks et al., 2019):

##### Conventional example 1

As shown in Table B^1^, the likelihood of recently (past 6 months) assisting injection drug use initiation was significantly related to recent enrollment in opioid agonist treatment (*z* = −2.52, *p* = 0.011), and methamphetamine injecting (*z* = 2.38, *p* = 0.017), in Vancouver. Enrollment in the opioid agonist treatment arm was associated with a lower likelihood of assisting injection initiation. The relative risk was significantly elevated for those injecting methamphetamine, whereas speedball injecting was not significantly associated with initiation assistance (*z* = 1.84, *p* = 0.064).

This example starts with a reference to a table. It then indicates the patterns of significance and the direction of the effects. The parameter estimates that describe the magnitude and functional form can be extracted from the table (see Marks et al., 2019); however, significance tests are the focus of what is being communicated. The parameter estimates are absent from the text. No information about the non-significant association is developed. Abandoning significance tests and broadening the focus to include parameter estimates increases both the total information content and information density of the text. Now we will rewrite this paragraph in the POCS style.

The first sentence can be a direct answer to the research question proposed. Based on prior evidence, we had hypothesized opioid agonist treatment enrollment would decrease the likelihood of assisting an initiation; thus, for our new first sentence, we propose:

Results of our multivariable model are consistent with our hypothesis that recent enrollment in opioid agonist treatment was associated with a decreased likelihood of recently assisting injection initiation in Vancouver.

We have begun by directly addressing how our findings answer our research question. Next, we want to present the details of the quantitative patterns. This can also be the first sentence when the functional association is simple. We also want to make sure to present the results in a way that increases the value of information available to the reader – in this case, instead of presenting regression point estimates, we present the relative risk and proportional effects. As such, we propose:

*Recent opioid agonist treatment enrollment was associated with a 12 to 63% reduction in likelihood of assisting initiation (RR: 0.58 95% CI: 0.37–0.88, p* = *0.011, Table B).*

This lets the reader know not only that we have a high degree of confidence in the direction of the effect (both indicated by the confidence interval and *p*-value), but also that the magnitude of the effect warrants further consideration that opioid agonist treatment should be considered as a tool for addressing injection initiation. If, for example, our confidence interval had been 0.96–0.98, even though we feel confident in the direction of the effect, we may deem it inappropriate to suggest changes to treatment implementation as a result based on such a small potential return on investment. Relying solely upon significance testing to determine the value of findings could result in the glossing over this critical piece of information (i.e., the effect size). In addition, we note that we have now included a reference to the table where further details and context can be inspected.

Finally, we want to examine additional patterns in the data. In the SOCS style paragraph, we reflected on the significance of both the effect of recent methamphetamine injection and recent speedball (heroin and cocaine) injection. While our primary research question focused on the impact of opioid agonist treatment, we can still also present results for related secondary questions regarding methamphetamine and speedball injection, so we write:

*Recent methamphetamine injection was associated with a 12% to 227% increase in likelihood of assisting initiation (RR: 1.91 95% CI: 1.12–3.27, p-value* = *0.017). Similarly, recent speedball injection was associated with an effect ranging from a 3% decrease to a 193% increase in likelihood of assisting initiation (RR: 1.68 95% CI: 0.97–2.93, p* = *0.064).*

Here, we find that methamphetamine and speedball injection had similar confidence interval estimates. Instead of saying that the impact of speedball injection was “not significant” where the *p*-values exceed.05, we present the confidence interval of methamphetamine and speedball injection relative risks. From this, the reader can evaluate if our conclusion that the findings preclude the possibility that speedball may have at most a small protective effect on assisting initiation. We can gain some knowledge from non-significant findings. Relevant stakeholders may determine that a 3% reduction to a 193% increase in risk is strong enough evidence to allocate resources to further study and/or intervene on speedball injection. We note that assessing the acceptability of characterizing an effect in this way that did not meet traditional standards of significance is a complex task and that it will be dependent on the consensus of the authors, reviewers, and editors. This subjectivity of assessment exemplifies the importance of the POCS style, as it requires all stakeholders in the peer-review process to engage critically with the interpretation of “not significant” findings.

Our new POCS paragraph reads:

*Results of our multivariable model were consistent with our hypothesis that recent enrollment in opioid agonist treatment was associated with a decreased likelihood of recently assisting injection initiation in Vancouver. Recent opioid agonist treatment enrollment was associated with a 12 to 63% reduction in likelihood of assisting initiation (RR: 0.58 95% CI: 0.37–0.88, p* = *0.011, Table B). Recent methamphetamine injection was associated with a 12% to 227% increase in likelihood of assisting initiation (RR: 1.91 95% CI: 1.12–3.27, p-value* = *0.017). Similarly, recent speedball injection was associated with an effect ranging from a 3% decrease to a 193% increase in likelihood of assisting initiation (RR: 1.68 95% CI: 0.97–2.93, p* = *0.064).*

#### Example 2: Associations Among Adolescent Alcohol Use, Expectancies, and School Connectedness

Using data from a community survey of high school students, we investigated the relationship among drinking expectancies, school connectedness and heavy episodic drinking (Cummins et al., 2019). Student perceptions of acceptance, respect, and support at their schools are reported to be protective against various risky health behaviors, including drinking. We wanted to know if the association was contingent on alcohol expectancies. Alcohol expectancies are cognitions related to the expected outcomes that a person attributes to drinking (Brown et al., 1987). In this study, higher expectancies indicate the respondent expects the outcomes of consuming alcohol to be more rewarding.

##### Conventional example 2

**FIGURE 1:**
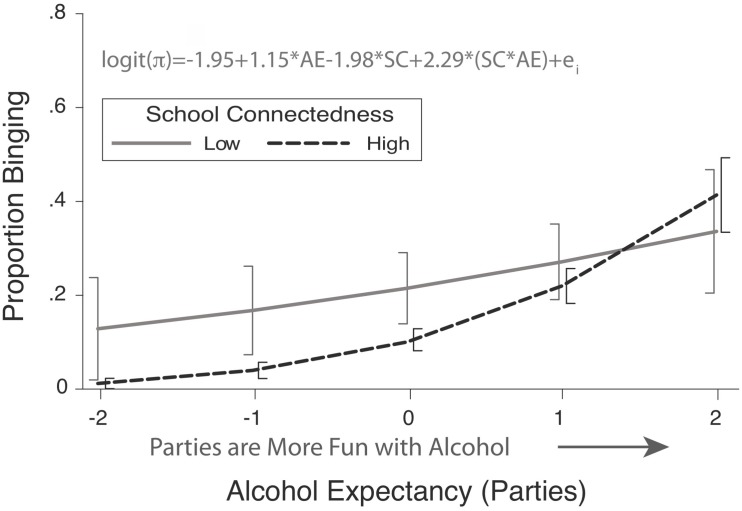
Modeled proportion of high school students engaging in heavy episodic drinking (binging) as a function of school connectedness (SC) and party-related alcohol expectancies (AE). Error bars represent 95% confidence intervals. Low and high school connectedness are at the minimum and maximum observed school connectedness, respectively. Modified from Cummins et al. (2019).

Figure 1 and Table 2^2^ depict the associations among recent (past 30 days) binge drinking, school connectedness, and alcohol expectancies. The model for recent (past 30 days) binge drinking with school connectedness, party-related alcohol expectancies, and their interaction as independent variables was statistically significant (Likelihood Ratio χ2 (3) = 171, *p* < 0.0001). Significant moderation was observed (OR = 9.89, SC X pAE interaction: *z* = 2.64, *p* = 0.008). The prevalence of binging was significantly higher for students reporting the highest expectancies as compared to those reporting the lowest expectancies when students also reported the highest school connectedness (*z* = 9.39, *p* < 0.001). The predicted prevalence of binge drinking was 17.9 times higher among students with the highest expectancies, as compared to those with the lowest expectancies. This same comparison was non-significant, where school connectedness was at its lowest (*z* = 1.84, *p* = 0.066).

While this is not an archetypal version of the SOCS style, its primary focus is on the patterns of significance. Some information is presented on the magnitudes of the associations in the text; however, metrics of estimation uncertainty are absent, as is information on the non-significant patterns. Much of the text is redundant with the tables or is unneeded (Cummins, 2009). For example, the first sentence is a simple reference to a figure with no indication of what the authors extracted from their inspection of the figure. The text itself does not help answer the scientific question. The functional associations and their magnitudes are not described, so there is no value added by the presence of the sentence. The latter sentences primarily function to identify statistically significant patterns. The measure of association strength is presented for one of the features of the model, which was statistically significant. The uncertainty of the estimate and the features were not described. Finally, it remains unclear how the results answer the substantive question under study. Now, we will rewrite this paragraph in the POCS style.

The first sentence needs to be a direct answer to the substantive question under study. We expected students who reported higher party-related expectancies (i.e., had more positive views of attending parties) would report higher odds of recent binge drinking. Further, we wanted to assess if school connectedness moderated this relationship. For our new first sentence, we propose:

We found that higher levels of alcohol expectancies were associated with greater odds of binge drinking and that this relationship was attenuated among those reporting the lowest level of school connectedness.

Here, we have directly answered our research question. Results of moderation analyses can be challenging to parse, exemplifying the need to clearly articulate how the results reflect upon the question under study is particularly important. This first sentence helps the reader navigate the more complex statements about contingent effects. We do this by presenting a data visualization and highlighting the relationship of party-related expectancies and binge drinking at the highest and lowest levels of school connectedness in the text, as such:

*For students reporting the highest level of school connectedness, the modeled odds of binge drinking was 30.5 (95% CI: 15.4–60.2) times higher for students with the highest level of alcohol expectancies as compared to the those with the lowest. This pattern was attenuated for students reporting the lowest levels of school connectedness (OR* = *3.18), such that the confidence interval for the modeled odds ratio ranged from 1.18 to 10.6 (95% CI) (Figure 1).*

Here, we have provided the reader two key pieces of information that quantifies our initial qualitative statement: first, for both students with the lowest and highest levels of school connectedness, we are confident there is a positive relationship between alcohol expectancies and binge drinking; and, second, that this relationship is attenuated amongst those with the lowest levels of school connectedness, as indicated by their non-overlapping confidence intervals. We have also provided the reference to Figure 1, reducing the initial examples’ 20-word directional sentence, to two words.

We also focus upon estimation uncertainty by presenting the confidence intervals in the body of the text. We direct the reader to recognize the lower bound of the for the odds ratio was near 1.18 for students with the lowest school connectedness. This can be returned to in the discussion. It could be pointed out that it is plausible alcohol expectancies are not strongly associated with binge drinking for these students. Deploying interventions targeting expectancies among these students could be an inefficient use of resources. Thus, getting an improved estimate of the effect size could be valuable to practitioners before committing to a rigid plan for deploying intervention resources. Not only should authors present measures of uncertainty (e.g., confidence intervals, credibility intervals, prediction intervals), they should base their interpretations on those intervals.

Finally, we want to reflect on additional patterns in the data. In the SOCS example, we presented the significance of the model fit, as well as the significance of the interaction effect. We provide additional information for the reader to assess the magnitude of the interaction. We give the reader a way to gauge this by contrasting the association at the extremes of school connectedness. We note that, in cases where the models are complex, word limits and a disposition toward being concise will force authors to be selective about which features are to be verbalized. Patterns of lower importance may not be described in the text but should be accessible to the reader through tables and figures.

Here, we also note that the reader should be able to evaluate the authors’ descriptive choices in the text and ensure those are faithful to the overall patterns. On the flip side of the coin, the author’s selections also initially guide the reader through the answers to the study’s questions that are supported by the content within the tables and figures. For reviewers and editors assessing works in the POCS style, it will be important to assess if the authors’ descriptive choices are faithful to the overall patterns of the results. This requires that authors provide adequate information in their tables and figures for reviewers to make such an assessment.

As a result, our new POCS paragraph reads as follows:

*We found that higher levels of alcohol expectancies were associated with greater odds of binge drinking and that there was evidence that the strength of this relationship was contingent on school connectedness, such that it was attenuated among students reporting the lowest level of school connectedness. For students reporting the highest level of school connectedness, the modelled odds of binge drinking was 30.5 (95% CI: 15.4–60.2, Figure 1 and Table 2) times higher for students with the highest level of alcohol expectancies as compared to the those with the lowest. This association was attenuated for students reporting the lowest levels of school connectedness (OR* = *3.18), such that the interval estimate of modelled odds ratio ranged from 1.18 to 10.6 (95% CI).*

## Discussion

We present a communication style that abandons the use of bright-line significance testing. By introducing the POCS style as a formal structure for presenting results, we seek to reduce barriers faced by researchers in their efforts to follow recommendations for abandoning the practice of declaring results statistically significant (Amrhein et al., 2019; Wasserstein et al., 2019). The examples provided demonstrate how the adoption of this general approach could help improve the field by shifting its focus during results generation to the simultaneous and integrated consideration of measures of effect and inferential statistics. Reviewers should also recognize that the use of POCS is not an indicator of statistical naivety, but rather one of a differing view on traditional approaches–this paper can be a useful resource for explaining POCS to unfamiliar reviewers. Writing results without the word “significant” is completely counter to the training and experience for most researchers. We hope that these examples will motivate researchers to attempt to draft their results without using or reporting significance tests. Although some researchers may fear that they will be left with a diminished ability to publish, this need not be the case. If the research findings do not stand up when described in terms of the functional associations, perhaps that research is not ready to be published. Indeed, with greater recognition of the replication crisis in the psychological sciences, we should pay more attention to the design features and basic details of the patterns of effects.

Significance testing should not be used to reify a conclusion. Fisher (1935) warned that an “isolated record” of a significant result does not warrant its consideration as a genuine effect. Although we want our individual works to be presented as providing a strong benefit to the field, our confidence that individual reports will hold is often unwarranted. We may benefit from cautiously reserving our conclusions until a strong and multi-faceted body of confirmatory evidence is available. This evidence can be compiled without bright-line significance testing. Improved reporting, that presents a full characterization of the functional relationships under study, can help to facilitate the synthesis of research generated knowledge into reviews and metanalyses. It is also consistent with American Psychological Association reporting standards, which promotes the reporting of exact *p*-values along with point and interval estimates of the effect-size (Appelbaum et al., 2018).

The strongest support for some of our research conclusions have been obtained from Bayesian probabilities based on informative priors (e.g., Cummins et al., 2019). This point serves to highlight the general limitations of focusing on frequentist based NHST in scientific research and the benefit of gauging evidence other substantive features, such as the design, explanatory breadth, predictive power, assumptions, and competing alternative models (de Schoot et al., 2011; Trafimow et al., 2018). The POCS is compatible with a more integrated approach to the valuation of research reports, whereas the continued use of bright-line significance testing is not (Trafimow et al., 2018).

We suspect that the quality of many papers will increase through the application of POCS. In part, this will be driven by a change in orientation toward the aims of research reports where the emphasis on the establishment of the presence of an association is substituted with an emphasis on estimating the functional form (magnitude, shape, and contingencies) of those relationships. The examples from our own work demonstrate that there should be no barrier to drafting papers with POCS. Research-based on an integrated examination of all statistical metrics (effect sizes, *p*-values, error estimates, etc.) shall lead to more meaningful and transparent communication and robust development of our knowledge base. Research findings should not be simply dichotomized – the quantitative principle that the categorization of a continuous variable will always lead to a loss of information also applies to *p*-values (Altman and Royston, 2006). In this paper, we provide examples of different ways to apply that principle.

## Author Contributions

Both authors listed have made a substantial, direct and intellectual contribution to the work, and approved it for publication.

## Conflict of Interest

The authors declare that the research was conducted in the absence of any commercial or financial relationships that could be construed as a potential conflict of interest.

## Footnotes

1 This reference is to Table B in the supplement to Marks et al. (2019).

2 This reference is to Table 2 in the supplement to Cummins et al. (2019).
